# Preventing Microbial Growth in Game Meat by Applying Polyphenolic Extracts from Olive Mill Vegetation Water

**DOI:** 10.3390/foods13050658

**Published:** 2024-02-22

**Authors:** Caterina Altissimi, Rossana Roila, David Ranucci, Raffaella Branciari, Dongjie Cai, Peter Paulsen

**Affiliations:** 1 Department of Veterinary Medicine, University of Perugia, 06121 Perugia, Italy; caterina.altissimi@studenti.unipg.it (C.A.); rossana.roila@unipg.it (R.R.); raffaella.branciari@unipg.it (R.B.); 2 College of Veterinary Medicine, Sichuan Agricultural University, Chengdu 611130, China; dongjie_cai@sicau.edu.cn; 3 Centre for Food Science and Veterinary Public Health, Clinical Department for Farm Animals and Food System Science, University of Veterinary Medicine Vienna, 1012 Vienna, Austria; peter.paulsen@vetmeduni.ac.at

**Keywords:** hydroxytyrosol, tyrosol, microbiology, food hygiene, wild boar, deer

## Abstract

We studied the efficacy of different formulations of polyphenol extracts (mainly containing hydroxytyrosol and tyrosol) from olive mill vegetation water on the microflora on the surfaces of game meat cuts with high or low initial bacterial loads. Meat with a high microbial load (>5 Log cfu/g; mean value = 6.83 ± 0.45 standard deviation) was immersed for 10 or 60 sec into 25% and 10% solutions of microencapsulated freeze-dried and non-encapsulated polyphenolic extracts. Aerobic colony, *Enterobacteriaceae*, *Pseudomonas* spp., and lactic acid bacteria counts were determined on treated samples compared to controls after 7 days of storage (in vacuum-packed conditions at +3 °C). Significant differences were registered only for aerobic colony count for a 10% liquid extract treatment (0.64 log reduction). In contrast, the dipping or immersion of game meat with low initial microbial loads (<5 Log cfu/g; mean value = 3.58 ± 0.72 standard deviation) in 10% solutions of the polyphenol extracts effectuated significant reductions in all bacteria counts (*p* < 0.002) at 7 and 14 days of storage for different extracts, independently from the application methods. The use of the extracts to inhibit bacterial growth in game meat should only be considered if a good hygienic baseline is guaranteed.

## 1. Introduction

The interest in natural preservatives in meat and meat product manufacturing has increased, especially regarding those derived from food industry by-products [1]. Because of their antioxidant and anti-inflammatory qualities, polyphenols are plant secondary metabolites that are increasingly being utilized in food, beverages, and innovative cosmetic formulations [2,3]. They are also employed in nutraceutical supplements [4]. Furthermore, in the last decade, numerous studies have described the effects of polyphenols obtained from plant extracts on foodborne pathogens and spoilage bacteria [5,6,7,8], both in vitro and in meat models. A few studies have also reported on the antimicrobial effects of specific polyphenols, preventing bacterial growth when directly applied to fresh meat or added to minced meat preparations [9,10]. Furthermore, the synergistic or antagonistic interactions of phenolic compounds with multiple other chemicals may influence the antibacterial properties of plant extracts [11]. Polyphenols are also evaluated after being mixed with other molecules or adsorbed onto coating agents [12,13]. Different polyphenols are present in olive leaves and fruits, and their amounts change according to geographical localization, cultivar, and season [14,15]. Although the olive fruit contains a high concentration of phenolic compounds, only 2% of them are found in the oil phase. The majority are lost in the solid pomace residue (about 45%; approximately 2–8 g of polyphenols/kg depending on processing) and the aqueous phase (approximately 53%) [16]. Large amounts of water (0.6–1.3 m^3^/1000 kg of processed olives) are added during the widely used three-phase extraction systems’ olive oil production process [17], which leads to the production of over 30 million m^3^ of oil mill vegetation water (OMVW) worldwide [18]. OMVW is a dark, mildly acidic liquid with high conductivity that is obtained from mechanically processing olives during the production of olive oil and contains a wide range of polyphenols, such as secroiridoids (oleuropein), simple phenols (hydroxytyrosol; tyrosol, 4-hydroxyphenyl acetate), phenolic acids (chlorogenic acid; vanillic acid; caffeic acid; p-coumaric acid; ferulic acid; and verbascoside), and flavones (luteolin, apigenin), in concentrations of 1–10 g/L [18]. Therefore, it can be used for polyphenol extraction. 

Polyphenol extracts from olive mill vegetation water (OMVW) have been investigated for their potential applications and effects, both in vitro [19,20] and in fresh meats, mainly in pork sausages and beef patties [19,21,22,23] but also chicken [24,25]. Despite having the same by-product origin, different compounds can be extracted, with potentially different antioxidant and antimicrobial activities [26]. Recently, specific preparations obtained from OMVW containing mainly hydroxytyrosol and tyrosol (without secroiridoids) are available on the market for application in food production. However, to date, no studies have reported on the antimicrobial effects of such compounds on game meat. 

Due to its ability to meet a growing number of demands from conscious consumers, hunted wild game meat is becoming more and more popular in the last decades. Consumer requirements include positive nutritional aspects regarding fat and protein content and quality [27], the careful consideration of consumer health aspects such as the avoidance of antibiotics and pharmaceuticals [28], and the ethical treatment of animals during the entire manufacturing process [29]. Indeed, game meat is sourced from animals free to live in a natural setting, not reared in intensive farming systems, and not subject to the stress of live transport or slaughtering [27,29]. Nevertheless, some concerns may arise regarding the hygiene level and safety of game meat [30]. Game meat is peculiar with respect to the way that it undergoes a different production process and is more prone to microbial contamination than meat from slaughtered farm animals [31,32]. This may be due to the mode of killing, i.e., a more or less accurate shot (e.g., involving the rupture of the gut or the exposure of damaged parts to the environment); an improperly performed in-field bleeding and evisceration process; delayed evisceration or a late onset of cooling [33,34,35]. The level of bacterial contamination is very important for fresh meat as it could affect its quality. The growth of spoilage microorganisms, together with endogenous enzymes and oxidation, can degrade various nutrients in meat and generate off-odors and off-flavors, as well as discoloration and slime, making the meat unfit for human consumption and generating waste [36]. For these reasons, the chemical antimicrobial treatment of hunted game carcasses, such as with lactic acid, has been suggested by different authors to be able to prevent bacterial growth during storage [32,37]. 

The aim of the study was to define the antimicrobial effect of different formulations of microencapsulated and non-encapsulated polyphenols obtained from OMVW on fresh game meat with different levels of contamination. Two series of trials were performed to assess the effects of the concentration and application method of polyphenolic extracts on meat hygiene indicators and spoilage microorganisms.

## 2. Materials and Methods

Two formulations of food-grade OMVW Polyphenolic Extract (PEs) already available on the market were considered in the trials. One was not encapsulated (in liquid state, LPE; Stymonphen liquid, Stymon, Patras, Greece; polyphenol content: 50,000 mg/kg; hydroxytyrosol/tyrosol ratio of 5:1 *w*/*w*), and one freeze-dried and encapsulated in maltodextrins (FPE; Stymonphen W50, Stymon, Patras, Greece; polyphenol content: 50,000 mg/kg; hydroxytyrosol/tyrosol ratio of 6:1 *w*/*w*). 

Fresh game meat (mainly from shoulder cuts) from wild boar (*Sus scrofa*), roe deer (*Capreolus capreolus*), and red deer (*Cervus elaphus*) was obtained from retailers in Austria. According to their records, the meat was from free-living wild game originating from Austria. The meat was kept vacuum-packaged at 3 ± 1 °C until the start of the trial. The muscles were cut into cubes measuring 2.5 × 2.5 × 2.5 cm.

Microbial counts were determined to assess the initial microbial loads. To this end, the samples were placed in sterile bags, and nine parts of Maximum Recovery Diluent (MRD) (Oxoid, Basingstoke, UK) were added. Homogenization of the sample was achieved using a Stomacher-type blender (Interscience, St. Nom, France); subsequently, serial tenfold dilutions were prepared in MRD. Samples were subjected to the following analyses: an aerobic colony count (ACC) performed according to ISO 4833-1:2013 [38] on Plate Count Agar (from Merck, Darmstadt, Germany) incubated for 72 h at 30 °C; enterobacteriaceae count (ENT) determined according to ISO 21528-2:2017 [39] on Violet Red Bile Glucose Agar (Merck) incubated for 24 h at 37 °C; *Pseudomonas* spp. (PSE) (Glutamate–Starch–Penicillin Agar (Merck) with Penicillin G supplement (Sandoz, Kundl, Austria) incubated for 72 h at 25 °C; and lactic acid bacteria (LAB) (on de Man Rogosa Sharpe Agar (Biolife Italiana, Milan, Italy) incubated for 48 h at 37 °C. The number of colony-forming units (cfu) per gram was converted to Log cfu/g. Two experiments were conducted.

### 2.1. Treatment of Game Meat with Initial High Microbial Loads (Experiment 1)

An initial trial was designed to determine the effect of the two different PEs on game meat with a high ACC load after 7 days of storage under refrigerated conditions. 

The pre-trial microbiological condition (T0) of the meat cubes was determined in 18 samples (6 from wild boar, 6 from red deer, and 6 from roe deer). The average values were 6.83 Log cfu/g (±0.45 standard deviation—sd) for ACC, 4.48 Log cfu/g (±0.44 sd) for ENT, 6.66 Log cfu/g (±0.43 sd) for PSE, and 4.44 Log cfu/g (±1.22 sd) for LAB.

The other 45 samples from the same muscles of the same subjects (15 from wild boar, 15 from red deer, and 15 from roe deer) were divided into five groups, with three replicates each: a control group (C) without any treatment, a group immersed for 1 min in a solution of 10% LPE (LPE10), a group immersed for 1 min in a solution of 25% LPE (LPE25), a group immersed for 1 min in a solution of 10% FPE (FPE10), and a group immersed for 1 min in a solution of 25% FPE (FPE25). After treatment, samples were allowed to dry for 5 min; then, they were vacuum-packaged (PA/PE film, Combivac90; Felzmann, Linz, Austria), and the packages were stored in refrigerated conditions (3 ± 1 °C) for 7 days, after which period the ACC, ENT, PSE, and LAB were determined as described previously.

Statistical analyses were performed using GLM SAS (SAS Institute, Cary, NY, USA) [40]. An ANOVA model was used to evaluate differences between C at T0 and T7. Another ANOVA model included PE formulation (C, LPE, and FPE) and concentration (10% and 25%) as fixed variables without a time effect, since that was available only for the C group. Animal species were not considered in the model as previous analyses had shown that species did not have a statistically significant effect. Tukey’s post hoc test was performed to evaluate the difference of the means. Statistical significance was established at *p* < 0.05.

### 2.2. Treatment of Game Meat with Initial Low Microbial Loads (Experiment 2)

A second trial was designed to determine the effect of application methods of 10% solutions of the two different PEs on game meat with low ACC load.

The microbial loads of 6 samples of wild boar meat were determined before the treatments (T0). 

A total of 45 samples were obtained from the same muscle, and the samples were randomly assigned to five groups: a control group (C) to which no treatments were applied; a group immersed for 1 min in a solution of 10% LPE (LPE I); a group dipped for 10 s in a solution of 10% LPE (LPE D); a group immersed for 1 min in a solution of 10% FPE (FPEI); and a group dipped for 10 s in a solution of 10% FPE (FPED). This trial was replicated three times. The average values of microbial counts at T0 (three replicates) were 3.58 Log cfu/g (±0.72 sd) for ACC, 1.88 Log cfu/g (±0.54 sd) for ENT, 2.20 Log cfu/g (±0.81 sd) for PSE, and 2.54 Log cfu/g (±0.72 sd). No differences were detected between replicates.

Samples were stored under vacuum under refrigerated conditions (3 ± 1 °C) for 7, 14, and 21 after which period, ACC, ENT, PSE and LAB counts were determined.

Statistical analyses were performed using the abovementioned software, and an ANOVA model was used to evaluate differences between C at T0 and T7, T14, and T21 and between C at T0 and PE groups at T7. Furthermore, another ANOVA model was used, with PE formulation (C, LPE, and FPE), method of application (I and D), and time (7, 14, and 21 days) serving as fixed variables. Replicates were not included as a factor in the model as no statistical differences were detected. Post hoc Tukey tests were therefore performed to evaluate the difference of the mean, and the difference was considered to be significant when *p* was <0.05.

## 3. Results

### 3.1. Treatment of Game Meat with Initial High Microbial Loads (Experiment 1)

In the C samples with high initial microbial loads, an increase in the ACC, ENT, and LAB was observed during the 7 days of observation. No difference was detected regarding the PSE counts.

The results obtained after 7 days of storage are reported in Table 1 for ACC, ENT, PSE, and LAB. For the ACCs, differences were recorded only between C and LPE10 and were below 1 Log cfu/g (Table 1). No differences were recorded for ENT, PSE, and LAB with values over 6 Log cfu/g for PSE and LAB and over 4.5 Log cfu/g for ENT.

### 3.2. Treatment of Game Meat with Initial Low Microbial Loads (Experiment 2)

**Figure 1:**
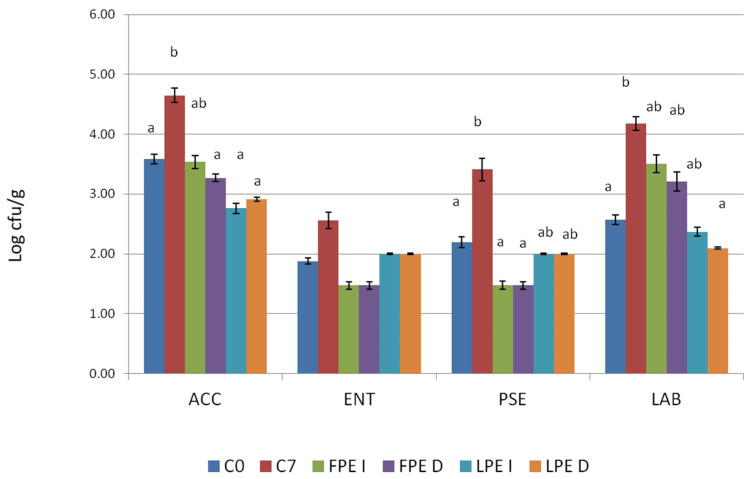
Difference of microbial loads (mean and standard error) between C samples before treatment and C, FPE I, FPE D, LPE I, and LPE D after 7 days of storage (Log cfu/g). Different letters for each microbial count (a,b) describe differences in the mean values (*p* < 0.05).

The results regarding the microbial loads of the C samples at T0 and T7 reveal an increase in all the parameters considered except for ENT. Indeed, no differences were recorded between C0 and FPEs and LPEs after 7 days of storage under vacuum-packaged and refrigerated conditions (Figure 1). 

The statistical analyses performed on the microbial loads (Table 2) showed significant effects of PE application and time on the microbial growth of all the bacteria populations considered. No significant difference was detected between the average microbial load values according to the method used for PE application (immersion or dipping), as the *p* values were always over 0.05. Time significantly affected the ACC and LAB microbial loads independently from the PE type and methods used. Regarding ENT, a significant increase was registered only between the C and FPE I groups, while for PSE, a significant difference was only detected between the C with the FPE D group (Table 2). 

The effect of the PEs on the C samples was significant for ACC after 7 days, with the exception of FPE I, and after 14 days only for FPE D. For ENT, differences were registered after 14 days between C and FPE I and FPE D and after 21 days between C and LPE I. The PSE counts were different between C and FPE I and FPE D at 7 and 14 days of observation. The LAB count was higher in C than LPE D after 7 days and in FPE D after 14 days. No differences were detected at 21 days between the C and PE groups with respect to ACC, PSE, and LAB. ENT at 21 days was lower in the LPE I group than in the C group.

## 4. Discussion

The first trial confirmed that game meat can be highly contaminated just after the handling and butchering processes. In this case, values even higher than 7 log per g or cm^2^ could be found [33]. The microbial loads in these meats increased under cold-storage conditions of +3 °C and under vacuum to final concentrations in the same order of magnitude as in meat cuts with low initial contamination but stored for 21 days.

For meat cuts with high initial bacterial loads, the effect of PEs on the microflora is limited or absent, even when high concentrations of PEs (25%) are used, and no decontamination of the meat is observed. Indeed, polyphenols are more likely to exert bacteriostatic rather than bactericidal effects, reducing the growth of some microbial populations during storage. The possible mechanisms of action proposed for similar compounds are the depletion of ATP inside the bacteria due to polyphenols binding ATP synthetase and altering the microbial metabolism [41]. Furthermore, polyphenols also cause the depolarization of bacterial cells with cell morphology modification, resulting in damage to the cell membrane and the leakage of cytoplasm [42,43]. According to other studies, tyrosol suppresses the activity of cyclooxygenase enzymes, and hydroxytyrosol can cause protein denaturation [44]. All these mechanisms could be responsible for the increase in the bacterial lag phase and the reduction in the exponential growth phase (log phase) detected in different bacterial populations [10,23]. It is possible that when a high number of bacteria were present in the meat, all the mechanisms that interfere with bacterial adaptation to the environment could not be overcome. The effectiveness of compounds with bacteriostatic activity may be restricted in these circumstances.

For an improvement of PE efficacy in food preservation, other possible technologies or antimicrobial substances could be combined in the food industry (i.e., vacuum packaging, high-pressure processing, bacteriocins, polysaccharides, and additives) [45,46,47]. PEs, as additional hurdles, combined with traditional and innovative preservation technologies require further investigation with respect to game meat.

When the initial microbial loads were below 4 Log cfu/g, the 10% PE solutions seemed to affect microbial growth. Indeed, for some of the groups tested, a delay in ACC, PSE, and LAB growth was registered after 7 days and remained to some extent until 14 days (i.e., the ACC in groups C and FPE D). At 21 days, when the microbial load reached high concentrations, the effects were no longer apparent. Other authors have reported a delay in microbial growth when OMVW polyphenols were used, but comparing data from the literature is challenging due to differences in the compounds present in the extracts, their concentrations and the application method used, and the meat matrix under study (e.g., minced meat preparations). The inhibition of bacterial growth was induced by the incorporation of polyphenol extracts from olive leaves and various by-products from the olive oil process into raw or cooked ground meats [10,23,48]. Fasolato et al. [24] reported a reduction in *Enterobacteriaceae* and *Pseudomonas* counts in chicken breast fillets dipped in a crude extract containing a total concentration of phenols of about 22 g/kg, whose main compounds were oleuropein aglycone, hydroxytyrosol, tyrosol, and verbascoside. 

The results of experiment 2 reveal that a delay in ENT growth was more evident after 14 and 21 days of storage, differing from the results concerning chicken meat reported by Fasolato et al. [24]. This could be due to differences in the meat considered and the PEs used, particularly when the extracted compounds exerted synergic effects. This phenomenon is still under debate. Some authors suggest that antimicrobial in vitro activity is best assessed by using purified molecules [49,50] with dose-dependent inhibition effects, also depending on the culture media adopted [51]. Other authors mention potential synergic effects between phenolic compounds or with other molecules [41,52], but further studies are needed on raw meat. 

Dipping for a short time (10 s), adopted by Fasolato et al. [24], was still sufficient for achieving bacteriostatic effects, regardless of whether PEs were in liquid or powder form. Encapsulation in maltodextrin after freeze-drying does not seem to affect the efficacy of the extract.

The results confirm the possible use of these PEs on game meat to prevent microbial growth, with a double impact on food sector sustainability, either via reducing food wastage due to microbial spoilage or due to meat oxidation or via reusing an olive-oil-by-product, which is an environmental pollutant necessitating specific disposal strategies [53]. Nonetheless, the relevance of the results should be discussed with respect to other aspects such as the effects on meat quality and regulatory considerations. Since polyphenol extracts are already available on the market as food-grade “flavouring”, their application, depending on the concentration, should have an impact on the sensorial characteristics of the products. Furthermore, the antioxidant activity of these compounds has to be taken into consideration when sensory attributes are investigated since they could be used to prevent the production of an off-color, -odor, and -taste due to the oxidation of meat during storage [54]. Other authors reported that acceptable sensory traits could be obtained by using 7% hydroxytyrosol formulations from OMVW in chicken frankfurters [55]. No sensory effects were reported for PEs from olive leaves (100 and 150 μg of phenols/g meat) in raw minced beef [56], and an improvement in the visual quality of cooked beef burgers was obtained with the use of OMVW PEs (87.5 mg of phenols/kg of meat) [57]. Furthermore, undesired side-effects of PEs on quality attributes could be mitigated by incorporating PEs in coating materials [58]. For this reason, polyphenols from olive byproducts have been used in different types of food packaging, such as polyethylene terephthalate/polypropylen (PET/PT); chitosan + glycerol; alginate + gelatin + glycerol; pectin-fish gelatin + glutaraldehyde +glycerol; κ-carrageenan + glycerol; multilayer polyethylene; and polyvinyl alcohol [59]. This is probably the most promising application of these PEs in the food industry, but it has been reported at the laboratory scale only, for limited kinds of foods, and not for game meat. Indeed, a deeper analysis of all the conditions affecting PEs’ efficacy with respect to the microbial spoilage of game meat, but also the effects on game meat quality attributes, is still needed.

## 5. Conclusions

The use of OMVW PE to inhibit bacterial growth in game meat should be considered only if a good hygienic baseline is guaranteed. When treatment was applied to highly contaminated meat, no consistent effect was found. This underpins EU considerations, where the prevention of microbial contamination and growth is of primary importance, whereas decontaminating treatments are only adjunct measures [60]. When low bacterial contamination occurred, a beneficial effect was evident in game meat stored under vacuum and refrigerated conditions. In these circumstances, the use of PEs could be favorable. Further studies are needed to consider the application of PEs to carcasses after evisceration, with the skin on (i.e., to treat body cavities), or to freshly exposed meat surfaces after the skinning of a carcass as an early-stage treatment with potential beneficial effects for the meat cuts taken from the so-treated carcasses. In addition, it would be appropriate to evaluate the effects of treatment on meat quality characteristics, particularly their antioxidant effect in meats that are more prone to oxidation than those of other animals. Furthermore, combinations with other preservatives or treatments need further evaluation.

## Figures and Tables

**Table 1:** Microbial counts (Log cfu/g) of game meat samples from experiment 1 (high initial ACC loads) after 7 days of storage.

Group		ACC	ENT	PSE	LAB
C		7.84 ^b^	5.12	6.97	7.05
LPE10		7.20 ^a^	4.84	6.53	6.69
LPE25		7.66 ^ab^	4.91	6.59	6.97
FPE10		7.44 ^ab^	4.77	6.47	6.60
FPE25		7.73 ^ab^	4.65	6.62	6.95
SEM		0.140	0.199	0.152	0.166
*p* value	PE	0.010	0.568	0.632	0.266
	Concentration	0.082	0.573	0.212	0.917
	PE × Con	0.328	0.250	0.766	0.456

n = 9 per experimental group. ACC = aerobic colony count; ENT = Enterobacteriaceae count; PSE = Pseudomonas count; LAB = Lactic acid bacteria count; C = control group; LPE10 = 10% solution of liquid polyphenolic extract; LPE25 = 25% solution of liquid polyphenolic extract; FPE10 = 10% solution of freeze-dried polyphenolic extract; FPE25 = 25% solution of freeze-dried polyphenolic extract. Different letters in the same column (a,b) describe difference in the mean values (*p* < 0.05).

**Table 2:** Microbial loads (Log cfu/g) of game meat samples from experiment 2 (low initial ACC loads) after 7, 14, and 21 days of storage.

Group	Time	ACC	ENT	PSE	LAB
C	7 days	4.65 ^aV^	2.56 ^a^	3.29 ^W^	4.18 ^aW^
	14 days	6.55 ^bY^	3.31 ^bV^	3.71 ^X^	6.07 ^bX^
	21 days	7.16 ^b^	5.33 ^bY^	4.54	6.78 ^b^
FPE I	7 days	3.53 ^aVW^	1.47	1.48 ^V^	3.51 ^aVW^
	14 days	5.90 ^bXY^	1.47 ^W^	1.65 ^Y^	4.96 ^bXY^
	21 days	6.41 ^b^	2.93 ^XY^	2.76	6.07 ^b^
FPE D	7 days	3.27 ^aW^	1.47 ^a^	1.48 ^aV^	3.21 ^aVW^
	14 days	4.75 ^bX^	1.47 ^aW^	1.47 ^aY^	4.41 ^bY^
	21 days	7.23 ^c^	4.19 ^bXY^	3.72 ^b^	7.04 ^c^
LPE I	7 days	2.76 ^aW^	1.99	2.00 ^VW^	2.37 ^aVW^
	14 days	5.43 ^bXY^	1.99 ^VW^	2.00 ^XY^	4.87 ^bXY^
	21 days	6.63 ^b^	2.58 ^X^	2.58	6.54 ^c^
LPE D	7 days	2.91 ^aW^	1.99	1.99 ^VW^	2.10 ^aV^
	14 days	5.20 ^bXY^	2.10 ^VW^	1.99 ^XY^	4.75 ^bXY^
	21 days	6.22 ^b^	3.11 ^XY^	2.36	6.15 ^b^
SEM		0.411	0.494	0.529	0.451
*p* value	PE	<0.001	<0.001	<0.001	0.002
	Method	0.471	0.160	0.651	0.689
	Time	<0.001	<0.001	<0.001	<0.001
	PE × T	0.238	0.023	0.014	0.203
	PE × M	0.946	0.572	0.417	0.588
	M × T	0.320	0.151	0.595	0.554
	PE × T × M	0.160	0.671	0.302	0.346

n = 9 per experimental group for each replicate. ACC = aerobic colony count; ENT = Enterobacteriaceae count; PSE = Pseudomonas count; LAB = lactic acid bacteria count; C = control group; LPEI = sample immersed for 1 min in a 10% solution of liquid polyphenolic extract; LPE D = sample dipped for 10 s in a 10% solution of liquid polyphenolic extract; FPE I = sample immersed for 1 min in a 10% solution of freeze-dried polyphenolic extract; FPE D = sample dipped for 10 s in a 10% solution of freeze-dried polyphenolic extract; PE = polyphenolic extract; T = time; M = method of application of the polyphenolic extract. In each column, different small letters (a,b,c) within the same group denote statistically significant differences in the mean values between times of storage (*p* < 0.05); likewise, different capital letters (V,W,X,Y) indicate significant differences in the mean values between groups (*p* < 0.05) for the same storage time.

## Data Availability

The original contributions presented in the study are included in the article, further inquiries can be directed to the corresponding author.
